# Concentrated Growth Factor Induces ER Stress and Apoptosis by Increasing Ceramide Generation in Selected Tumour Cell Lines

**DOI:** 10.1111/jcmm.70916

**Published:** 2025-10-24

**Authors:** Andrea Palermo, Francesco Spedicato, Anna Giudetti, Daniele Vergara, Franco Ferrante, Alessandro D'amuri, Laura Giannotti, Benedetta Di Chiara Stanca, Christian Demitri, Fabrizio Damiano, Eleonora Stanca, Luisa Siculella

**Affiliations:** ^1^ Department of Experimental Medicine (DiMeS) University of Salento Lecce Italy; ^2^ Department of Biological and Environmental Sciences and Technologies (DiSTeBA) University of Salento Lecce Italy; ^3^ Private Practice Lecce Italy; ^4^ National Research Council (IPCB‐CNR) Institute of Polymers, Composites and Biomaterials Naples Italy

**Keywords:** apoptosis, autophagy, breast cancer, ceramide, CGF, ER‐stress, osteosarcoma

## Abstract

Concentrated growth factor (CGF), a blood‐derived autologous biomaterial, is increasingly utilised in regenerative medicine and, recently, in cancer‐related surgeries. Rich in cytokines, platelets, nucleated cells and fibrin scaffolds, CGF offers therapeutic promise but requires rigorous safety evaluation in oncology. This study explores the effects of CGF‐conditioned medium (CGF‐CM) on breast cancer (MCF7, MDA‐231) and osteosarcoma (SaOS‐2, MG‐63) cell lines. Our findings reveal that CGF‐CM selectively induces cytotoxic effects in MCF7 and SaOS‐2 cells, while no cytotoxicity was observed in MDA‐231 and MG‐63 cells. Early apoptosis in MCF7 and SaOS‐2 cells was accompanied by mitochondrial dysfunction, evidenced by an increased BAX/BCL‐2 ratio and cytochrome c release. CGF‐CM treatment also elevated ceramide and triglyceride levels, linking lipid metabolic changes to cancer cell death. Endoplasmic reticulum (ER) stress markers, ATF6 and XBP1, were significantly upregulated in MCF7 and SaOS‐2 cells, highlighting the role of ER stress in CGF‐CM‐induced cytotoxicity. Furthermore, CGF‐CM inhibited autophagic flux, as demonstrated by altered LC3 and p62 protein levels, disrupting cellular homeostasis and contributing to apoptosis. These findings highlight the selective cytotoxic effects of CGF‐CM on specific cancer cell lines. The intricate interplay between mitochondrial dysfunction, ER stress, autophagy inhibition and lipid metabolism highlights its complex mechanisms of action.

## 1 Introduction

Surgery remains one of the primary methods for both curative and palliative treatment of many cancers. In recent years, the use of platelet‐rich plasma (PRP) and platelet‐rich fibrin (PRF) has been explored to enhance wound healing and minimise surgical side effects. Recent studies evaluated the effects of platelet derivatives on the growth and proliferation of tumor cells. The application of PRP at injury sites has shown a tendency to reduce complication rates without increasing local recurrence rates [1, 2]. It has been reported that PRP, in combination with OncoTherad (MRB‐CFI‐1), is promising for the treatment of non‐muscle invasive bladder cancer (NMIBC) by enhancing immune activation, leading to significant tumor progression inhibition while modulating the NMIBC microenvironment to a cytotoxic profile [3]. PRF also reduces tumor cell proliferation and promotes the expression of tumor‐suppressor genes in osteoblastic and fibroblastic cancer cell lines, suggesting a potential therapeutic role for PRF in treating localised tumors [4].

Additionally, platelet derivatives can activate death receptor pathways, promoting apoptosis only in cancer cells [5] or reducing tumor proliferation [6].

However, PRF stimulates proliferation and migration in oral squamous cell carcinoma cells, raising concerns about its use in certain cancer types [7].

The effects of platelet derivatives on cancer cell death are complex and highly context‐dependent, influenced by the specific cancer type, tumor microenvironment and the balance of growth factors and cytokines within the platelet derivatives.

Despite studies on the applications of platelet derivatives in cancer patients [1, 2, 8], there is still limited knowledge of their molecular effects on tumour cells.

Our previous studies showed that Concentrated growth factors (CGF), a third‐generation platelet derivative considered a modified form of PRF, facilitate the recruitment, proliferation and maturation of cells involved in bone regeneration. CGF is a fibrin scaffold containing a concentration of growth factors and cells higher than other autologous platelet concentrates [9, 10, 11]. The growth factors, such as platelet‐derived growth factor, transforming growth factor‐β1, fibroblast growth factor β, vascular endothelial growth factor (VEGF), insulin growth factor, endothelial growth factor and bone morphogenetic protein, are gradually released, ensuring controlled and sustained regenerative properties while reducing the risk of inflammatory responses associated with excessive concentrations of growth factors [12, 13, 14, 15]. CGF induces the activity of macrophages promoting THP‐1 monocyte/macrophage transition, creating a microenvironment conducive to tissue regeneration [15]. The release of anti‐inflammatory cytokines and growth factors in the injury site activates intracellular signalling pathways like the PI3K/AKT [15] and MAPK/ERK pathways [16], which promote cell survival, angiogenesis and regeneration [10, 14, 17].

Here we studied the effects of CGF on the viability of breast cancer (MCF7 and MDA231) and osteosarcoma (SaOS‐2 and MG‐63) cell lines. We observed that CGF induced apoptosis only in MCF7 and SaOS‐2 cells.

## 2 Results

### 2.1 CGF‐CM Can Affect Tumour Cell Viability and Migration

**FIGURE 1:**
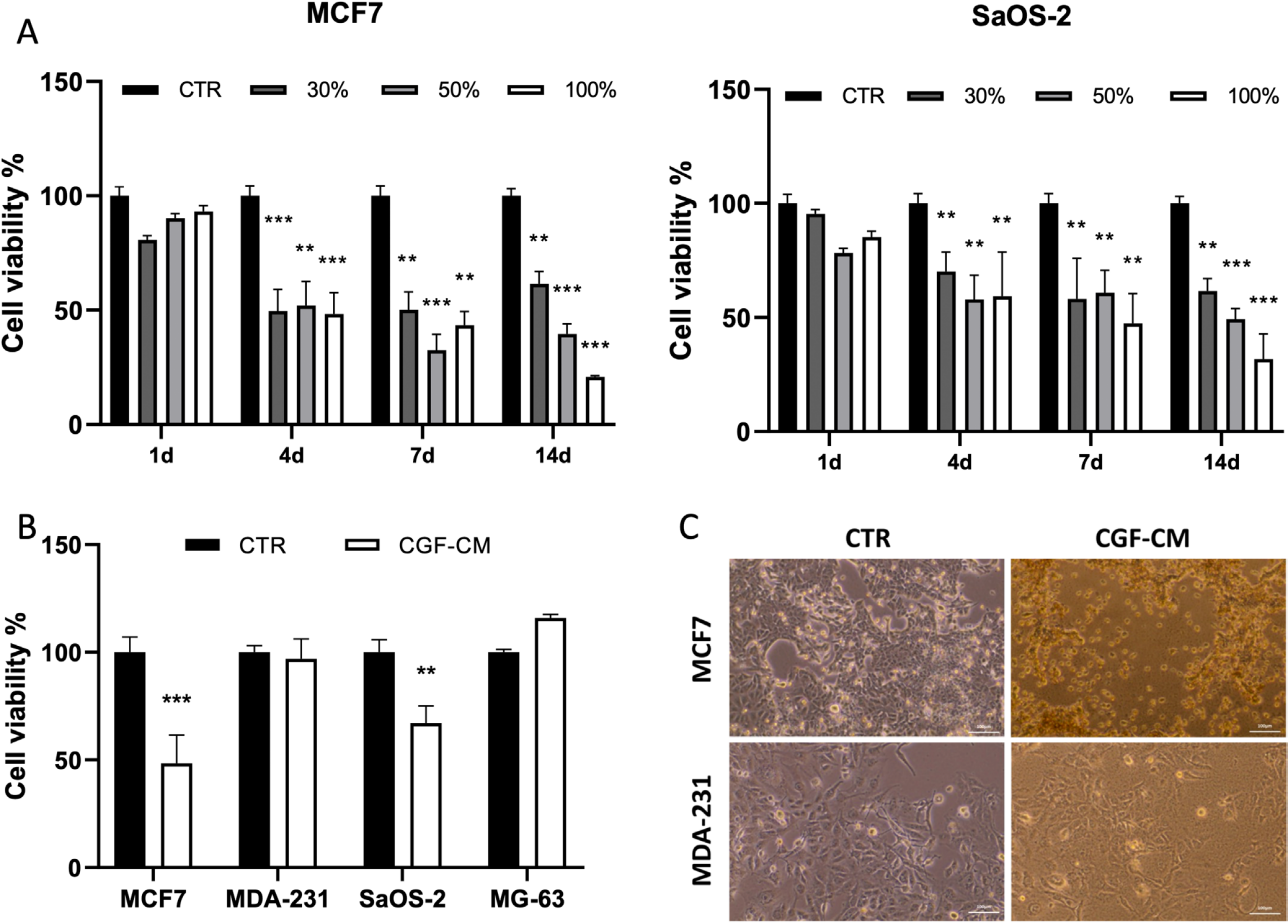
CGF‐CM induced cell death in specific tumour cell lines. (A) Cell viability was assessed by MTT assay. MCF7 and SaOS‐2 were treated with concentrations of CGF‐CM ranging from 30% to 100% for indicated times; (B) MCF‐7, MDA‐231, SaOS‐2 and MG‐63 cells were treated with 30% CGF‐CM for 4 days. Results were expressed as a percentage of the control cells for each cell line. Data are presented as the mean ± SD (*n* = 3) and experiments were repeated three times independently. **p < 0.01; ***p < 0.001 as compared to control for each cell line. (C) Phase‐contrast micrographs of cells treated with 30% CGF‐CM for 4 days. Images are representative of five independent experiments. Scale bar 100 μm.

MTT assay was performed to evaluate the effect of CGF‐conditioned medium (CGF‐CM) at different concentrations (30%, 50% or 100%) and for different times (1, 4, 7, 14 days) on MCF7, MDA‐231, SaOS‐2 and MG‐63 cell viability. Cells cultured in a medium without CGF‐CM represented the control experiment (CTR). When compared to CTR, CGF‐CM treatment did not affect MCF7 and SaOS‐2 cell viability on the first day. However, on the fourth day, the treatment reduced cell viability by approximately 50% at all concentrations compared with CTR (Figure 1A). MCF7 cell viability appeared strongly reduced by CGF‐CM concentrations higher than 50% at 14 days of treatment. Comparable results were observed performing the same experiment in SaOS‐2 cells with a slightly lower effect on cell viability reduction with respect to MCF7 cells (Figure 1A). Conversely, 30% CGF‐CM for 4 days did not reduce the viability of MDA‐231 and MG‐63 cells (Figure 1B); the same for other CGF‐CM concentrations and times (Figure S1A). CGF‐CM treatment did not modulate cell migration in all tumour cell lines (Figure S2). The reduction of cell viability was accompanied by evident alterations in morphology in MCF7 and SaOS‐2, but not in MDA‐231 and MG‐63 cells treated with 30% CGF‐CM for 4 days (Figure 1C, Figure S1B).

Since incubation with 30% CGF‐CM was found to be effective in reducing the viability of MCF7 and SaOS‐2 cells, this experimental condition was chosen to perform all the subsequent experiments.

### 2.2 CGF‐CM Induces Apoptosis on SaOS‐2 and MCF7 Cells

**FIGURE 2:**
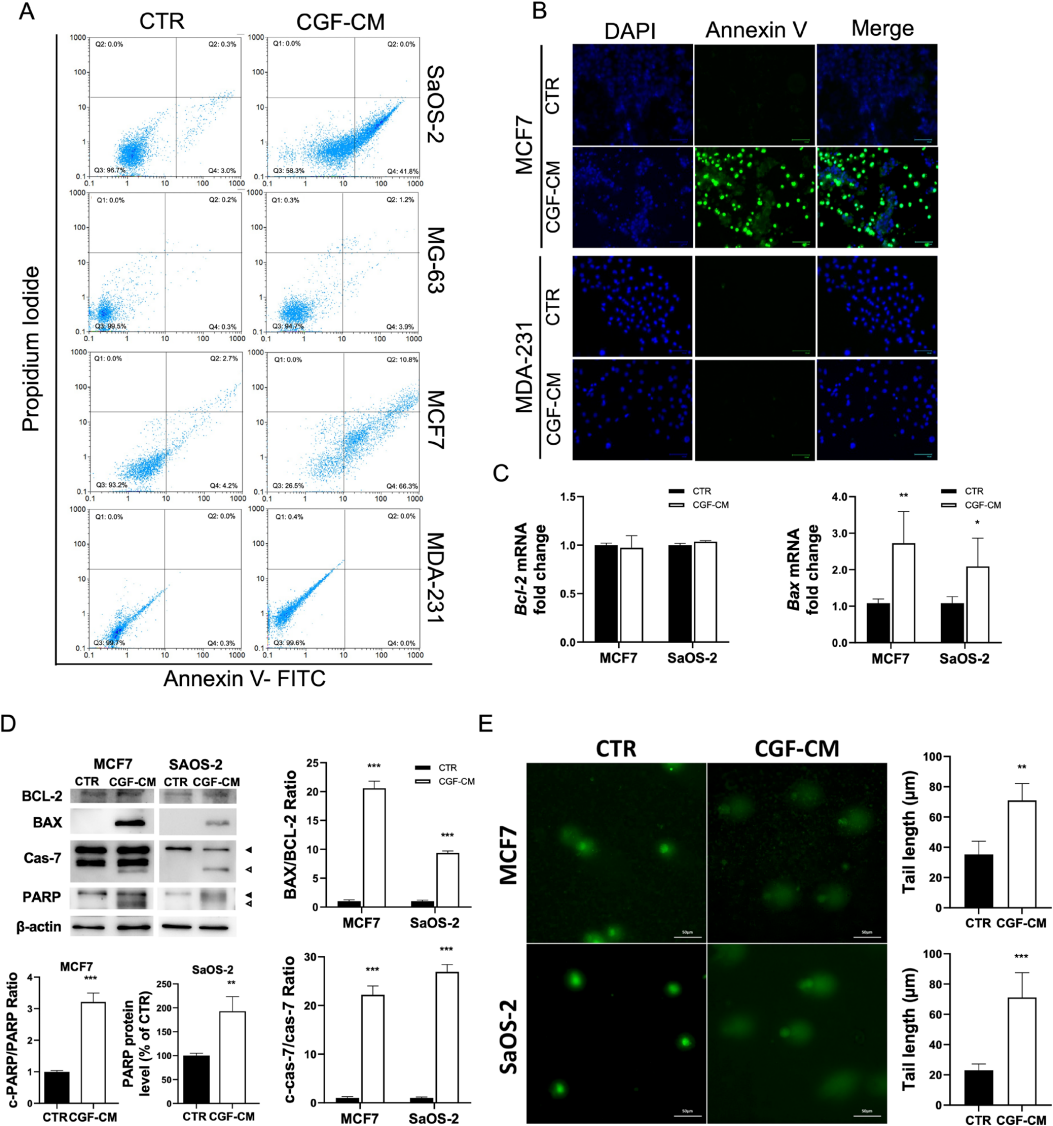
CGF‐CM induced apoptosis in MCF7 and SaOS‐2 cells. (A) Flow cytometry analysis of Annexin V and propidium iodide staining in untreated SaOS‐2, MG‐63, MCF7 and MDA‐MB‐231 cells (CTR) and treated with 30% CGF‐CM for 4 days. Q1 represents necrotic cells, Q2 late apoptotic cells, Q3 alive cells and Q4 early apoptotic cells. (B) Fluorescent micrographs of DAPI and Annexin V staining in MCF7 and MDA‐MB‐231 cells treated with 30% CGF‐CM for 4 days and CTR cells. (C) *Bax* and *Bcl‐2* mRNA were analysed by Real‐time PCR, quantified and reported in graphs as fold change with respect to CTR. (D) BCL‐2, BAX, full‐length and cleaved caspase 7 and full‐length and cleaved PARP levels were analysed by Western Blotting experiments. Densitometric quantification was performed, and the cleaved PARP/PARP ratio, cleaved caspase 7/pro‐caspase‐7 ratio, and BAX/BCL‐2 ratio were reported as fold change with respect to CTR. Black arrow, full‐length protein; white arrow, cleaved protein. (E) Comet assay was performed in untreated MCF7 and SaOS‐2 cells (CTR), and in the cells treated with 30% CGF‐CM for 4 days. For the evaluation of DNA damage, comet areas were quantified, and values were reported in the graph. The results were expressed as the mean ± SD of triplicate measurements from three independent experiments. *p < 0.05; **p < 0.01; ***p < 0.001 as compared to CTR for each cell line.

We further investigated the cytotoxic effects of CGF‐CM treatment by investigating the mechanism of cell death. Therefore, we examined apoptotic and necrotic cell populations by flow cytometry. The Annexin V/Propidium Iodide labelling assay revealed that after 4 days of 30% CGF‐CM treatment, about 60% of MCF7 and 50% of SaOS‐2 cells were apoptotic when compared to CTR. No significant changes in cell death were measured in MDA‐231 and MG‐63 (Figure 2A), as expected. This result was confirmed also by fluorescent microscopy images of Annexin V/DAPI staining in MCF7, MDA‐231 (Figure 2B) and SaOS‐2 and MG‐63 cells (Figure S3A).

Furthermore, the involvement of apoptosis in the CGF‐CM‐induced cell death was investigated by analysing key apoptosis markers. Compared to untreated cells, mRNA and protein levels of the anti‐apoptotic protein BCL‐2 did not change after CGF‐CM treatment in MCF7 and SaOS‐2. On the contrary, the treatment strongly increased the expression of the pro‐apoptotic protein Bax in MCF7 and SaOS‐2, as shown by Real‐time PCR and Western Blotting analysis (Figure 2C,D). Accumulation of cleaved caspase 7, the activated form, was detected in both cell lines (Figure 2D). Since poly(ADP‐ribose) polymerase (PARP) is a specific target of caspase‐7, the level of its full‐length and cleaved forms was analysed by Western Blotting in CTR and CGF‐CM treated cells. CGF‐CM treatment strongly increased PARP levels in MCF7 and SaOS‐2 cells (Figure 2D). However, cleaved PARP was only observed in MCF7 cells (Figure 2D). Another hallmark of apoptosis is fragmented DNA, which can be evaluated by comet assay. Our results showed that CGF‐CM treatment significantly increased comet tail length in MCF7 and SaOS‐2 cells with respect to CTR (Figure 2E). Conversely, the effect was not observed in MDA‐231 and MG‐63 cells (Figure S3B).

### 2.3 CGF‐CM Impairs Mitochondrial Function

**FIGURE 3:**
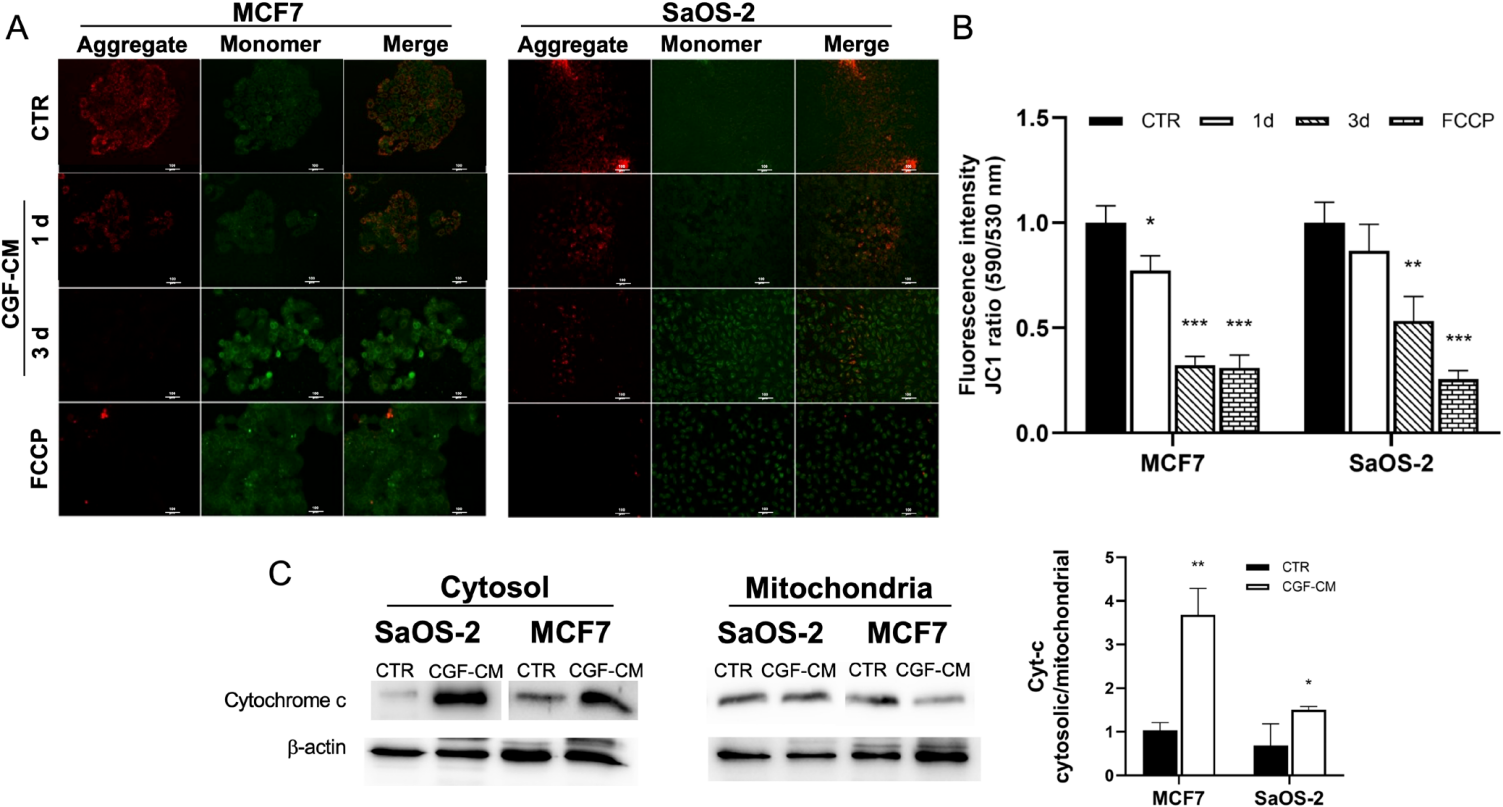
CGF‐CM treatment induced a decrease in mitochondrial membrane potential. (A) Change in mitochondrial membrane potential (ΔΨ_m_) was evaluated by JC1 staining assay in CTR, in cells treated with CGF‐CM for 1 and 3 days, and in cells treated with FCCP as a positive control. Scale bar, 100 μm. (B) Quantitative analysis of the ratio of aggregated (red) and monomeric (green) JC‐1 fluorescent intensity. (C) Cytochrome c protein level in cytosolic and mitochondrial protein extracts was measured in MCF7 and SaOS‐2 cells treated with 30% CGF‐CM for 4 days and in CTR. The content of cytochrome c was normalised vs. β‐actin. The results were expressed as the mean ± SD of triplicate measurements from three independent experiments. *p < 0.05; **p < 0.01; ***p < 0.001 as compared to CTR for each cell line.

To test whether CGF‐CM effects on reduced cell viability were associated with mitochondrial dysfunction, mitochondrial membrane potential ΔΨ_m_ was measured fluorometrically by JC‐1 staining assay after 1, 3 and 5 days of CGF‐CM treatment and compared to untreated CTR. ΔΨ_m_ alteration can be evidenced by the reduction of red fluorescent J‐aggregate and the increase of green JC‐1 monomer. Already after 1 day of treatment, a ΔΨ_m_ reduction of approximately 30% was observed in CGF‐CM treated MCF7 and SaOS‐2 compared to CTR, as indicated by the reduced J‐aggregate/JC‐1 ratio (red fluorescence/green fluorescence) (Figure 3A,B). The reduction of the J‐aggregate/JC‐1 ratio was more evident after 3 and 5 days of CGF‐CM treatment (Figure 3A,B). A similar decrease in membrane potential was observed by treating cells with FCCP, a depolarising agent used as a positive control (Figure 3).

Alteration of ΔΨ_m_ suggests a disruption of mitochondrial membrane integrity, which can be followed by cytochrome c leakage from the mitochondria. Therefore, cytochrome c protein levels in cytosolic and mitochondrial protein fractions were measured by Western Blotting analysis. Our findings indicated that, when compared to CTR, the level of cytosolic cytochrome c increased in MCF7 and, to a lesser extent, in SaOS‐2 cells treated with CGF‐CM (Figure 3C). The leakage of cytochrome c from mitochondria to the cytosol compartment was also supported by measuring the cytosolic cytochrome c/mitochondrial cytochrome c ratio, which was augmented in CGF‐CM treated cells compared with CTR.

### 2.4 CGF‐CM Modulates Lipid Metabolism and Increases Lipid Droplet Accumulation

**FIGURE 4:**
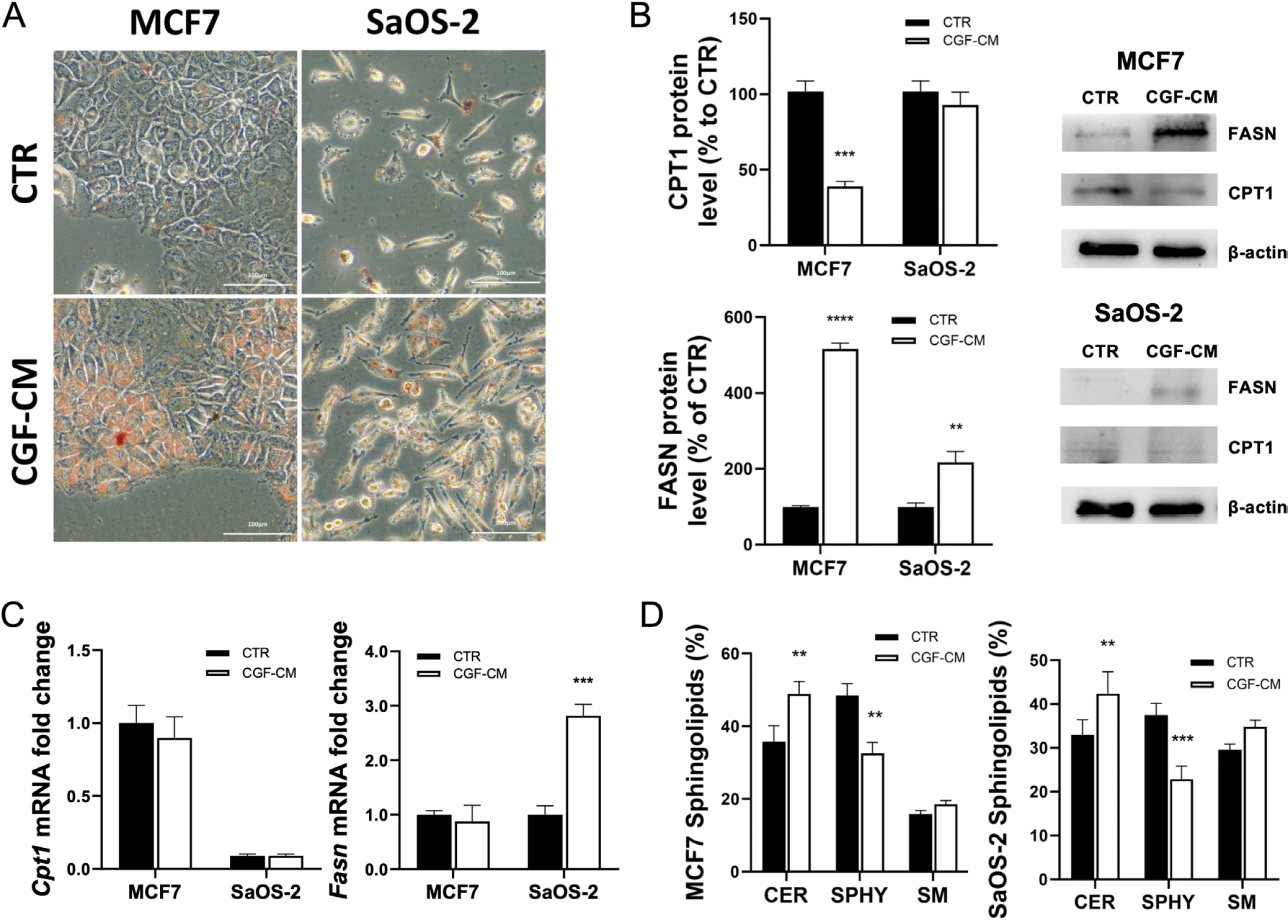
CGF‐CM increased lipid droplet amount in MCF7 and SaOS‐2 cells. (A) The micrographs depict the lipid droplet accumulation, visualised by Oil Red O staining, in cells treated with 30% of CGF‐CM for 48 h and control cells. The bars in micrographs correspond to 100 μm. Images are representative of three independent experiments. (B) Indicated cell lines were treated with 30% CGF‐CM or incubated in DMEM low glucose (CTR) for 48 h, and mRNA was quantified by Real‐time PCR. *Gapdh* was used as a housekeeping gene for normalisation. **p< 0.01; ***p<0.001; ****p< 0.0001. (C) Expression protein levels in total protein extracts from each cell line treated with 30% CGF‐CM or incubated in DMEM low glucose (CTR) for 48 h. The content of the respective protein was quantified by densitometric analysis and expressed as a percentage compared to own control cells. β‐actin was used for normalisation. Results are expressed as mean ± SD; experiments were repeated three times independently (*n* = 3). ***p < 0.001 as compared to CTR for each cell line. (D) Lipids were extracted from indicated cell lines treated with 30% CGF‐CM or incubated in DMEM low glucose (CTR) for 48 h. Then lipids were loaded on the silica gel for TLC analysis. Data are expressed as mean ± SD (*n* = 3) and reported as % of total lipid species. **p < 0.01, ***p < 0.001 compared to CTR for each cell line. CER = Ceramide; SM = sphingomyelin; SPHY = sphingosine.

The accumulation of intracellular lipids can trigger cellular stress, leading to the activation of apoptotic pathways and ultimately resulting in cell death. We explored the mechanisms of apoptotic cell death induced by CGF‐CM in MCF7 and SaOS‐2 cells by analysing lipid accumulation and the modulation of the expression of genes involved in lipid metabolic pathways by Oil Red O (ORO) staining, Real‐time PCR and Western Blotting experiments. Firstly, we observed a great increase in lipid droplet formation only in CGF‐CM‐treated MCF7 and SaOS‐2 cells, compared to CTR cells (Figure 4A). MDA‐231 cells were positive for ORO staining; however, no significant differences were observed in CGF‐CM‐treated cells when compared to CTR cells (Figure S4A). CTR and CGF‐CM‐treated MG‐63 cells did not show any ORO staining (Figure S4A). We investigated, in MCF7 and SaOS‐2 cells, the expression level of fatty acid synthase (FASN) and carnitine palmitoyltransferase‐1 (CPT‐1), key enzymes involved in lipogenesis and β‐oxidation, respectively. In MCF7 cells, CGF‐CM treatment did not modulate the mRNA level of both FAS and CPT‐1 when compared to CTR. On the contrary, FASN protein level increased by about 4‐fold and CPT‐1 decreased by about 0.6‐fold in CGF‐CM‐treated MCF7 compared to CTR cells. In SaOS‐2 cells, CGF‐CM supplementation induced a high increment of FASN mRNA and protein, whereas CPT‐1 mRNA and protein were weakly detected (Figure 4B,C). An opposite trend was observed in cells that did not die following CGF‐CM treatment. An increment of both metabolic gene expressions was observed in CGF‐CM‐treated MDA‐231 cells with respect to CTR. However, FASN protein level was drastically reduced, whereas a great increment of CPT‐1 protein level was observed in CGF‐CM‐treated cells with respect to CTR. In MG‐63 cells, CGF‐CM supplementation reduced mRNA and protein levels of FASN whereas CPT‐1 mRNA and protein levels were not detected (Figure S4B,C).

The increase in lipid synthesis could be linked to the ceramide production [18], a sphingolipid species found in cell membranes, able to regulate processes such as apoptosis, cell cycle and autophagy [19]. Sphingolipid composition was analysed by TLC. We found that CGF‐CM treatment did not change sphingomyelin levels but significantly increased ceramide while decreasing sphingosine levels in CGF‐CM‐treated cells in both MCF7 and SaOS‐2 cell lines with respect to CTR (Figure 4D).

### 2.5 CGF‐CM Induces ER Stress and Impairs Autophagy Flux in MCF7 and SaoS‐2

**FIGURE 5:**
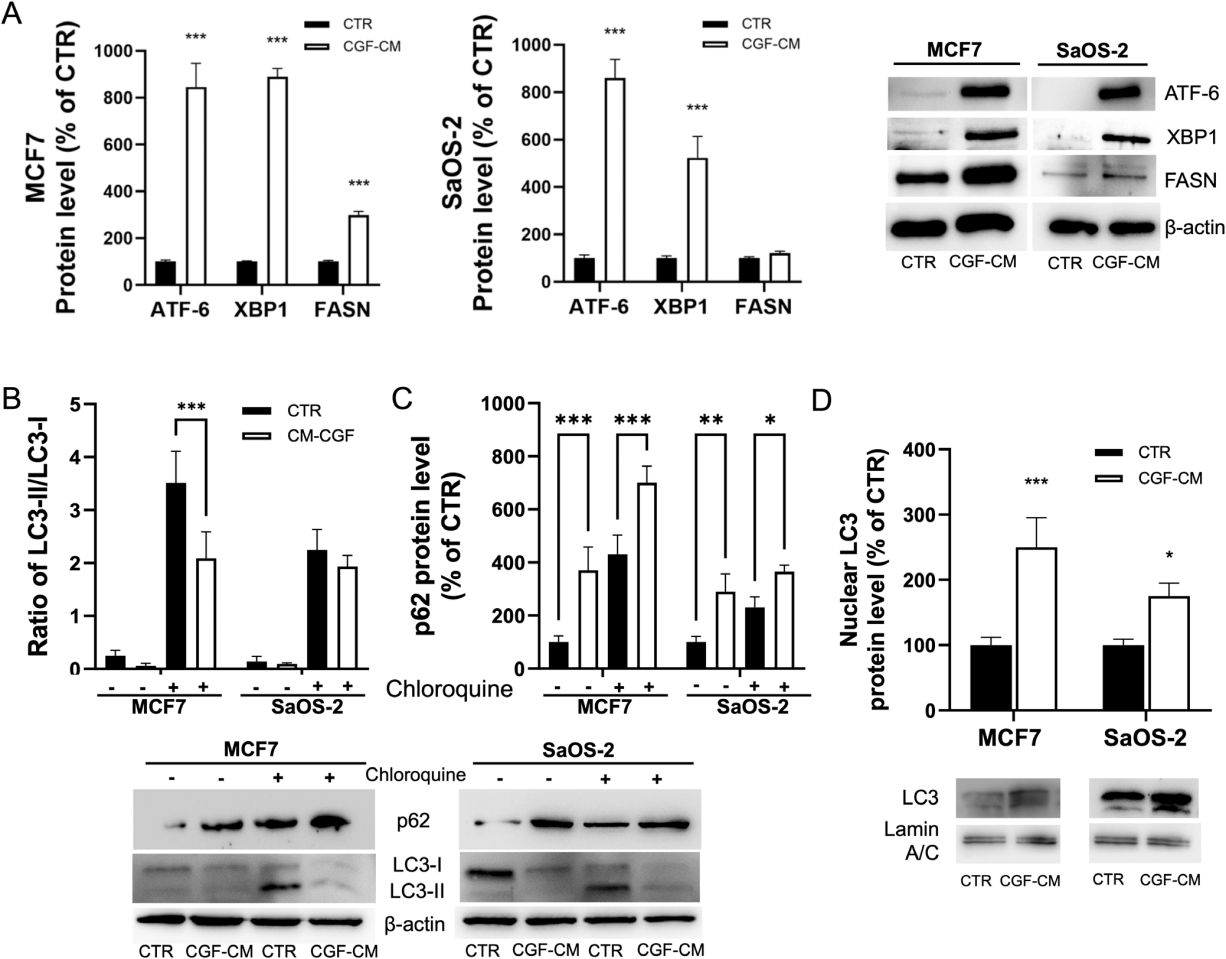
Effect of CGF‐CM treatment on autophagic flux. (A) ATF‐6, XBP1 and FASN protein levels in total protein extracts from each cell line treated with 30% CGF‐CM or incubated in DMEM low glucose (CTR) for 24 h. (B) LC3 protein expression levels in total protein extracts from each cell line treated with 30% CGF‐CM or incubated in DMEM low glucose (CTR) for 48 h. Densitometric quantification was performed, and the LC3‐II/LC3‐I ratio was calculated and reported in graphs as fold change with respect to CTR. (C) p62 protein level analysed by Western Blot. β‐actin was used as housekeeping. Results are expressed as mean ± SD. Experiments were repeated three times independently (*n* = 3). (D) LC3 protein level in nuclear protein extracts from MCF7 and SaOS‐2 cells treated with 30% CGF‐CM for 48 h and in CTR. The content of nuclear LC3 was normalised vs. laminin A/C. The results were expressed as the mean ± SD of triplicate measurements from three independent experiments. *p < 0.05; **p < 0.01; ***p < 0.001 as compared to CTR for each cell line.

To further investigate the cell death mechanism induced by CGF‐CM treatment on MCF7 and SaOS‐2 cells, the expression of ER‐stress and autophagy markers was investigated. ER‐stress pathways activation may precede alterations in autophagic flux and apoptosis. After 1 day of CGF‐CM treatment, XBP1 and ATF‐6 protein expression was strongly upregulated when compared to CTR. FASN protein level was increased in MCF‐7 treated cells, whereas no significant change was observed in CGF‐CM‐treated SaOS‐2 cells when compared to CTR (Figure 5A).

Key proteins involved in autophagy, such as LC3 and p62 [20], provide insights into the regulation of autophagic flux under experimental conditions. No significant differences in the ratio of LC3‐II/LC3‐I were observed in CGF‐CM‐treated cells with respect to CTR. However, CGF‐CM treatment induced a decrease in LC3‐I protein (Figure 5B) and an increment in p62 protein level when compared to CTR (Figure 5A). In the presence of chloroquine, the LC3‐II/LC3‐I ratio decreased in CGF‐CM‐treated cells when compared to CTR (Figure 5B). Furthermore, LC3 protein level increased in the nuclei of treated cells compared to CTR. These results supported the hypothesis that CGF‐CM treatment inhibited autophagy before autolysosome formation [21, 22].

## 3 Discussion

CGF and other platelet derivatives have shown promise in enhancing wound healing across various surgical fields, including dentistry, cosmetic surgery and orthopaedics. Studies indicate that CGF can induce cell migration, proliferation [11, 15] and, in certain conditions, cell differentiation [13, 14]. However, the effects of platelet derivatives on cancer cells are highly context‐dependent and vary according to the type of cancer, microenvironment and concentration of growth factors. Research suggests that growth factors in platelet derivatives may promote cancer cell proliferation and migration, particularly in cancers driven by growth factor receptors, such as specific types of breast and oral squamous cancer [7, 23]. For instance, the high levels of VEGF in PRF could enhance angiogenesis in tumours, potentially contributing to their growth and metastasis. Conversely, other studies suggest that PRF and CGF may inhibit proliferation and induce cancer cell death, particularly through the modulation of apoptotic and necrotic pathways [8, 17, 24]. The application of platelet derivatives in oncological surgery is spreading; thus, it requires clear information on their effects on the proliferation of tumour cells. In this study, we analyse the effect of CGF‐CM on the viability and apoptosis induction of two breast cancer cell lines (MCF7 and MDA‐231) and two osteosarcoma cell lines (SaOS‐2 and MG‐63).

Our findings revealed that CGF‐CM significantly reduced the viability of MCF7 and SaOS‐2 cells after 4 days of treatment at concentrations ranging from 30% to 100%, with 30% CGF‐CM being the lowest effective concentration. Consequently, 30% CGF‐CM was used to perform all the experiments. Annexin V/PI assay indicated that CGF‐CM treatment induced early apoptosis in MCF7 and SaOS‐2 cells, a result supported by DNA damage observed in comet assay images. It is important to underline that the cytotoxic effects of CGF appeared specific to MCF7 and SaOS‐2 cells; indeed, no cytotoxicity was observed in MDA‐231 and MG‐62. Additionally, CGF‐CM treatment did not affect the proliferation or migration of MG‐63 and MDA‐231 cells. The treatment increased the lipid metabolic enzyme expression in MDA‐231, even if no variation in the total lipids was observed with respect to CTR. Although this aspect may require further investigation, neither DNA damage nor an increase in invasive capacity were observed in MDA‐231 and MG‐63 cells. Reduction of viability or cytotoxicity was not observed also in mesenchymal stem cells [13], fibroblasts [25] or endothelial cells [14] treated with CGF.

DNA damage accumulates in apoptosis following PARP inactivation. PARP is a DNA repair enzyme leading to excessive energy consumption. Caspase‐3 and caspase‐7 are responsible for PARP cleavage, thereby preventing energy depletion and necrosis while promoting apoptosis. MCF7 does not express caspase 3 [26], so we analysed the level of caspase‐7. CGF treatment induced caspase‐7 activation in both MCF‐7 and SaOS‐2 cells. Unexpectedly, while PARP cleavage was observed only in MCF7 cells, SaOS‐2 cells showed an increase in total PARP protein levels with no detectable cleavage. This finding aligns with recent findings indicating that osteosarcoma cells lacking PARP cleavage can still undergo apoptosis [27]. In recent years, PARP inhibitors have been tested as an effective treatment in osteosarcomas since high levels of the PARP protein are associated with a poor prognosis [28]. Cell viability data suggest that SaOS‐2 cells could be more resistant to CGF‐CM treatment than MCF7; indeed, the percentage of dead cells recorded in MCF7 after 4 days of CGF‐CM treatment was observed in SaOS‐2 after 7 days.

However, we observed that CGF‐CM treatment increased the BAX/BCL‐2 ratio and promoted cytochrome c release from mitochondria in both MCF7 and SaOS‐2 cells, supporting the involvement of the intrinsic apoptotic pathway. BAX is an antiapoptotic protein involved in the mitochondrial permeabilisation with other BCL2 family proteins; indeed, early apoptosis is accompanied by a reduction in ΔΨ_m_ due to the opening of pores in the mitochondrial membrane.

CGF treatment induced the accumulation of neutral lipids and triglycerides in MCF‐7 and SaOS‐2 cells specifically. Lipid accumulation was supported by an increase in FASN mRNA and protein levels, a key enzyme in lipogenesis. Furthermore, we observed a significant rise in ceramide levels in CGF‐CM‐treated MCF‐7 and SaOS‐2 cells, accompanied by a decrease in sphingosine. Ceramides are bioactive sphingolipids known to inhibit cell proliferation and promote apoptosis in response to cellular stress [29]. Ceramides can also play a critical role in mitochondrial permeabilisation by forming pores with Bax in the mitochondrial membrane, leading to cytochrome c release from the intermembrane space into the cytosol [30, 31, 32], the loss of mitochondrial membrane potential and the cleavage of PARP [33, 34]. The increase in ceramide can occur through the activation of neutral sphingomyelinase or ceramide synthase in the de novo synthesis pathway. The increased ceramide levels in this study suggest that CGF‐CM treatment may enhance ceramide synthesis, potentially via the de novo or salvage from sphingosine pathways [35].

In cancer cell models, ceramide synthase activity has been linked to apoptosis induction [30, 36, 37]. Triglyceride accumulation can similarly contribute to mitochondrial dysfunction and apoptosis, as reported in *Atgl* ^−/−^ macrophages, where ceramide generation leads to these effects [38]. Previous studies have linked ceramide [39] and lipid accumulation [40] to ER stress and inhibition of autophagic flux. Our data reveal that CGF‐CM induced ER stress within the first day of treatment, as demonstrated by elevated levels of ATF6 and XBP1 proteins in MCF7 and SaOS‐2 cells. XBP1, a transcription factor, is known to regulate ER stress response genes as well as genes involved in lipogenesis [41]. Notably, in MCF7 cells, CGF‐CM treatment increased FASN expression, a XBP1 target, while no significant changes in *Fasn* mRNA were observed in SaOS‐2 cells within 1 day of treatment. However, both FASN protein and mRNA levels increased in SaOS‐2 cells after 2 days of CGF‐CM treatment.

ER stress pathway is linked with the alteration of autophagic flux [42]. Our findings indicate that CGF‐CM treatment inhibited autophagic flux in MCF7 and SaOS‐2 cells, as evidenced by increased p62 and decreased LC3 protein levels. Comparable results were obtained in *Atg5* ^−/−^ murine embryonic fibroblasts [43]. LC3 can localise in the nucleus and then it can move into the cytosol after deacetylation catalysed by Sirtuin1 upon induction of autophagy [44]. Interestingly, we observed that LC3 protein levels increase in the nuclei of CGF‐CM‐treated cells compared to CTR. We hypothesise that CGF treatment induced cellular stress that increased nuclear LC3 levels impairing autophagy; a similar effect has been demonstrated in colorectal cancer cells after knockout of *Atg7* [45].

The impaired autophagic flux impacts cellular homeostasis and survival, leading to mitochondrial dysfunction and apoptosis, as autophagy plays a crucial role in the removal of damaged mitochondria [18, 46].

In conclusion, our findings demonstrate that CGF selectively induced apoptosis in MCF7 and SaOS‐2 cells. In detail, CGF activated ER stress pathways which induced activation of lipogenic genes, and it may be linked to an increase in ceramide amount. These events impaired autophagy and mitochondrial function, activating the intrinsic apoptosis pathway. Further research is needed to elucidate the molecular mechanism of these effects and to assess the potential therapeutic implications of CGF in cancer treatment. Additionally, studies are needed to confirm the safety profile of CGF in other cancer types, as emerged from the results obtained on MDA‐231 and MG‐63 cells.

## 4 Materials and Methods

### 4.1 Preparation of CGF‐Conditioned Medium

Venous blood (8 mL) from 10 healthy, non‐smoking adult donors, aged between 27 and 50 years, was collected and immediately centrifuged by a Medifuge device (Medifuge MF200; Silfradent srl, Forlì, Italy), at 25°C, to obtain CGF clot, as previously described [12]. Informed consent was obtained from the donors included in this study in accordance with the Declaration of Helsinki. For each set of experiments, CGFs were prepared from the same blood sample of a single donor. To remove excess serum, the CGF clot was washed with phosphate‐buffered saline (PBS) and cultured in a sterile dish with 2 mL of Dulbecco's Modified Eagle Medium (DMEM) low glucose at 37°C in a humidified atmosphere with 5% CO_2_ for 14 days. Then, the CGF clot was removed, and the CGF‐conditioned medium (CGF‐CM) was collected and centrifuged at 12,000 rpm for 10 min. Then, the supernatant was stored at −80°C until use.

### 4.2 Cell Culture and Treatment

Human tumour cells MCF7, MDA‐231, SaOS‐2 and MG‐63 were purchased from ATCC (Rockville, MD, USA). The human cancer cell lines were cultured in high glucose DMEM (Euroclone, Milan, Italy) supplemented with 10% FBS (Euroclone, Milan, Italy), 100 U/mL penicillin and 100 μg/mL streptomycin at 37°C in an atmosphere of 5% CO_2_. The cells were maintained in an exponential growth phase during experiments. For treatment, confluent cells were shifted to the DMEM low glucose, supplemented with 10% FBS and subsequently treated with indicated concentrations of CGF‐CM for 1, 3 or 5 days. CGF‐CM concentrations were calculated based on the volume of CGF‐CM that was added to the total volume of the culture medium.

### 4.3 Cell Viability Assay

The MTT (3‐(4,5‐dimethylthiazol‐2‐yl)‐2,5‐diphenyltetrazolium bromide) assay was performed to assess the viability of cells after treatment with CGF‐CM. Cells were seeded in a 96‐well plate and treated with different concentrations of CGF‐CM (30%, 50% or 100%) for indicated times. Then, 20 μL MTT solution (10 mg/mL) was added to each well and incubated at 37°C for 4 h. Then, the MTT solution was removed and 100 μL of DMSO was added to solubilise MTT‐formazan crystals. The absorbance of the converted dye was measured at 570 nm using an iMark microplate reader (Bio‐Rad, Hercules, CA, USA).

### 4.4 Cell Apoptosis Measurement

The apoptotic cells were quantified using the Annexin V FITC kit (Miltenyi Biotec, Bergisch Gladbach, Germany) following the manufacturer's protocol. The cytofluorimetric analysis was performed with CyFlow space (Partec‐sismex, Kobe, Japan), and the data were analysed using FloMax software (version 2.3).

### 4.5 Comet Assay

After treatment, cells were harvested by trypsinisation, washed in PBS, counted and re‐suspended in low‐melting point agarose (Invitrogen, Carlsbad, CA, USA) to obtain a concentration of 0.5 × 10^6^ cells/mL. This solution was used to drop a droplet onto a microscope slide covered with a layer of normal‐melting point agarose. After 5 min at 4°C, the slides were immersed in lysis solution (2.5 M NaCl, 10 mM Tris HCl, 100 mM EDTA and Triton X‐100 1%) and incubated overnight at 4°C and then in alkaline buffer (300 mM NaOH and 1 mM EDTA) for 30 min at 4°C. After that, electrophoresis was performed at 20 V for 30 min at 4°C. Then the slides were gently washed with PBS and stained with Sybr Green (Life Technologies, Foster City, CA, USA) at a dilution of 1:2000 in PBS for 5 min in the dark. DNA damage was evaluated under a fluorescent microscope and DNA fragmentation was determined by measuring % Tail DNA [47] using ImageJ software (version 1.54f). At least 30 cells were counted in each of the three replicates.

### 4.6 Western‐Blot Analysis

Whole protein cell extracts of human tumour cells for Western‐blot analysis were obtained as previously described [13]. Separated proteins were then transferred electrophoretically onto a nitrocellulose membrane (Pall, East Hills, NY, USA). Equal protein loading was confirmed by Ponceau S staining. The filter was blocked with 2.5% (*w*/*v*) non‐fat dried milk in buffered saline. Blots were incubated overnight at 4°C with specific primary antibodies diluted 1:1000. The primary antibodies used were: Bax (sc‐7480), Bcl2 (sc‐7382), Cas‐7 (sc‐56063), PARP‐1 (SC‐8007), cytochrome c (sc‐13156), FASN (BD‐610963), CPT1 (sc‐393070), ATF6 (sc‐166659), XBP‐1 (sc‐7160), LC3A (Cell Signalling #4108), p62 (sc‐48402). Anti‐β‐actin (sc‐47778) and anti‐Lamin a/c (Cell Signalling #4777) were used for normalisation. The immune complexes were detected using appropriate peroxidase‐conjugated secondary antibodies and enhanced chemiluminescent detection reagent (Amersham International, Corston Bath, UK). Densitometric analysis was carried out on the Western blots by using the ChemiDoc MP Image System (BioRad, Segrate (Mi), Italy).

### 4.7 RNA Extraction and Real‐Time PCR

Total RNA was extracted from cells grown in a 35 mm ∅ culture dish using the Trizol (Sigma, Merck Life Science S.r.l., Milan, Italy) following the manufacturer's protocol. The reverse transcriptase reaction (20 μL) was carried out using 1 μg of total RNA, random primers and MultiScribe Reverse Transcriptase (Applied Biosystem, Monza, Italy) according to the manufacturer's protocol. Quantitative gene expression analysis was performed in a CFX Connect Real‐time System (BioRad, Segrate (Mi), Italy) using SYBR Green technology (FluoCycle‐Euroclone, Milan, Italy). Primers used in Real‐time PCR are reported in Table 1. The efficiency of each primer was tested using a standard curve in duplicate. The quantifications were performed using the ∆∆CT method, and the *Gapdh* gene was used as an internal control for normalizstion. The specificity of the PCR products was confirmed by the melting curve analysis.

**TABLE 1:** List of used primers.

Gene	Accession number	Sequences 5′–3′
*Gapdh*	NM_017008	F: GCATGGCCTTCCGTGTTCCTACC R: GCCGCCTGCTTCACCACCTTCT
*Bcl2*	NM_000633.3	F: TGGGATGCCTTTGTGGAACT R: GAGACAGCCAGGAGAAATC
*Bax*	NM_001291428.2	F: GATGCGTCCACCAAGAAGCT R: CGTCCACTCGGAAAAAGACC
*Cpt1*	NM_001031847.2	F:ACGGCCAACTGCATGTCC R:CATCCACCCGTGGTAGG
*Fasn*	NM_004104	F: CCTGCGTGGCCTTTGAAAT R: CATGTCCGTGAACTGCTGC

### 4.8 JC‐1 Dye for Mitochondrial Membrane Potential Measurements

Mitochondrial membrane depolarisation was detected by a shift in fluorescence emission of the lipophilic cationic probe 5,5′,6,6′‐tetrachloro‐1,1′,3,3′‐tetraethylbenzimidazolo‐carbocyanine iodide (JC‐1) (Santa Cruz Biotechnology, Dallas, Texas, USA). The cells were seeded in 24 well plates at a density of 1.5 × 10^4^ and treated under CTR and CGF‐CM conditions. After treatment, JC‐1 (2 μM) in fresh DMEM low glucose was added in each well, and the plate was incubated at 37°C with 5% CO2 for 15 min. Then, the absorbance of the monomer or aggregate dye was measured at 530 or 590 nm, respectively, using the microplate reader Biotek Cytation5 (Agilent technologies, CA, USA). The red/green ratio was used to assess differences in mitochondrial potential.

### 4.9 Analysis of Cell Lipids

After treatment, total lipids were extracted from an equal amount of MCF7 and SaOS‐2 cell proteins by the Bligh and Dyer procedure and loaded on silica gel plates for separation by thin‐layer chromatography (TLC). Plates were developed with toluene/methanol (70/30, v/v). Thereafter, plates were uniformly sprayed with 10% cupric sulfate in 8% aqueous phosphoric acid, allowed to dry for 10 min at room temperature, and then placed into a 145°C oven for 10 min [48]. The identification of ceramide, sphingosine and sphingomyelin was made by developing specific standards under the same experimental conditions. Band intensity was measured by densitometric analysis.

### 4.10 Statistical Analysis

Data were presented as mean ± standard deviation (SD) and analysed by Student's test or one‐way analysis of variance (ANOVA) followed by Tukey's multiple comparison test. The analysis was performed using GraphPad Prism 9 (GraphPad Software, USA), and *p* < 0.05 was considered statistically significant.

## Author Contributions

**Andrea Palermo:** conceptualization (equal), methodology (equal), resources (equal). **Francesco Spedicato:** formal analysis (equal), investigation (equal), visualization (equal), writing – original draft (equal). **Anna Giudetti:** investigation (equal), resources (equal), writing – review and editing (equal). **Daniele Vergara:** investigation (equal), methodology (equal). **Franco Ferrante:** resources (equal), validation (equal). **Laura Giannotti:** validation (equal), visualization (equal). **Benedetta Di Chiara Stanca:** validation (equal), visualization (equal). **Alessandro D'amuri:** data curation (equal), validation (equal). **Christian Demitri:** project administration (equal), resources (equal). **Fabrizio Damiano:** supervision (equal), validation (equal), writing – review and editing (equal). **Eleonora Stanca:** conceptualization (equal), investigation (equal), writing – original draft (equal). **Luisa Siculella:** project administration (equal), supervision (equal), writing – review and editing (equal).

## Consent

Written informed consent has been obtained from the donors to publish this paper.

## Conflicts of Interest

The authors declare no conflicts of interest.

## Supporting information


**Figure S1:** Cell viability was assessed by MTT assay.
**Figure S2:** Wound healing assay (scratch assay) in MCF7, MDA‐231, SaOS‐2 and MG‐63 cells treated with 30% CGF‐CM for and control cells.
**Figure S3:** (A) Fluorescent micrographs of DAPI and Annexin V staining in SaOS‐2 and MG‐63 cells treated with 30% CGF‐CM for 4 days and control cells. (B) Comet assay was performed in untreated MDA‐231 and MG‐63 cells (CTR), and cells treated with 30% CGF‐CM for 4 days.
**Figure S4:** (A) The micrographs depict the lipid droplet accumulation, visualised by Oil Red O staining, in cells treated with 30% of CGF‐CM for 48 h and control cell. (B) The bars in micrographs correspond to 100 μm. Images are representative of three independent experiments. Indicated cell lines were treated with 30% CGF‐CM or incubated in DMEM low glucose (CTR) for 48 h and mRNA was quantified by Real‐time PCR. Gapdh was used as a housekeeping gene for normalisation. Results are expressed as mean ± SD; experiments were repeated three times independently (*n* = 3). *p < 0.05 and ***p < 0.001 for each cell line compared with its own control. (C) Expression protein levels in total protein extracts of each cell lines treated with 30% CGF‐CM or incubated in DMEM low glucose (CTR) for 48 h. The content of the respective protein was quantified by densitometric analysis and expressed as percentage with respect to own control cells. β‐actin was used for normalisation. Results are expressed as mean ± SD; experiments were repeated three times independently (*n* = 3). *p < 0.01 and ***p < 0.001 for each cell line compared with its own control.

## Data Availability

All the data generated during the current study is included in the article. All data are available from the corresponding author on request.
