# Supplementary material for: Concentrated Growth Factor Induces ER Stress and Apoptosis by Increasing Ceramide Generation in Selected Tumour Cell Lines
Source: J Cell Mol Med. 2025 Oct 24;29(20):e70916. doi: 10.1111/jcmm.70916 (PMC12551144; doi:10.1111/jcmm.70916)
Supplement: Supplementary file 1 — Figure S1: Cell viability was assessed by MTT assay. Figure S2: Wound healing assay (scratch assay) in MCF7, MDA‐231, SaOS‐2 and MG‐63 cells treated with 30% CGF‐CM for and control cells. Figure S3: (A) Fluorescent micrographs of DAPI and Annexin V staining in SaOS‐2 and MG‐63 cells treated with 30% CGF‐CM for 4 days and control cells. (B) Comet assay was performed in untreated MDA‐231 and MG‐63 cells (CTR), and cells treated with 30% CGF‐CM for 4 days. Figure S4: (A) The micrographs depict the lipid droplet accumulation, visualised by Oil Red O staining, in cells treated with 30% of CGF‐CM for 48 h and control cell. (B) The bars in micrographs correspond to 100 μm. Images are representative of three independent experiments. Indicated cell lines were treated with 30% CGF‐CM or incubated in DMEM low glucose (CTR) for 48 h and mRNA was quantified by Real‐time PCR. Gapdh was used as a housekeeping gene for normalisation. Results are expressed as mean ± SD; experiments were repeated three times independently (n = 3). *p < 0.05 and ***p < 0.001 for each cell line compared with its own control. (C) Expression protein levels in total protein extracts of each cell lines treated with 30% CGF‐CM or incubated in DMEM low glucose (CTR) for 48 h. The content of the respective protein was quantified by densitometric analysis and expressed as percentage with respect to own control cells. β‐actin was used for normalisation. Results are expressed as mean ± SD; experiments were repeated three times independently (n = 3). *p < 0.01 and ***p < 0.001 for each cell line compared with its own control. [file JCMM-29-e70916-s001.pdf]

Supplementary materials

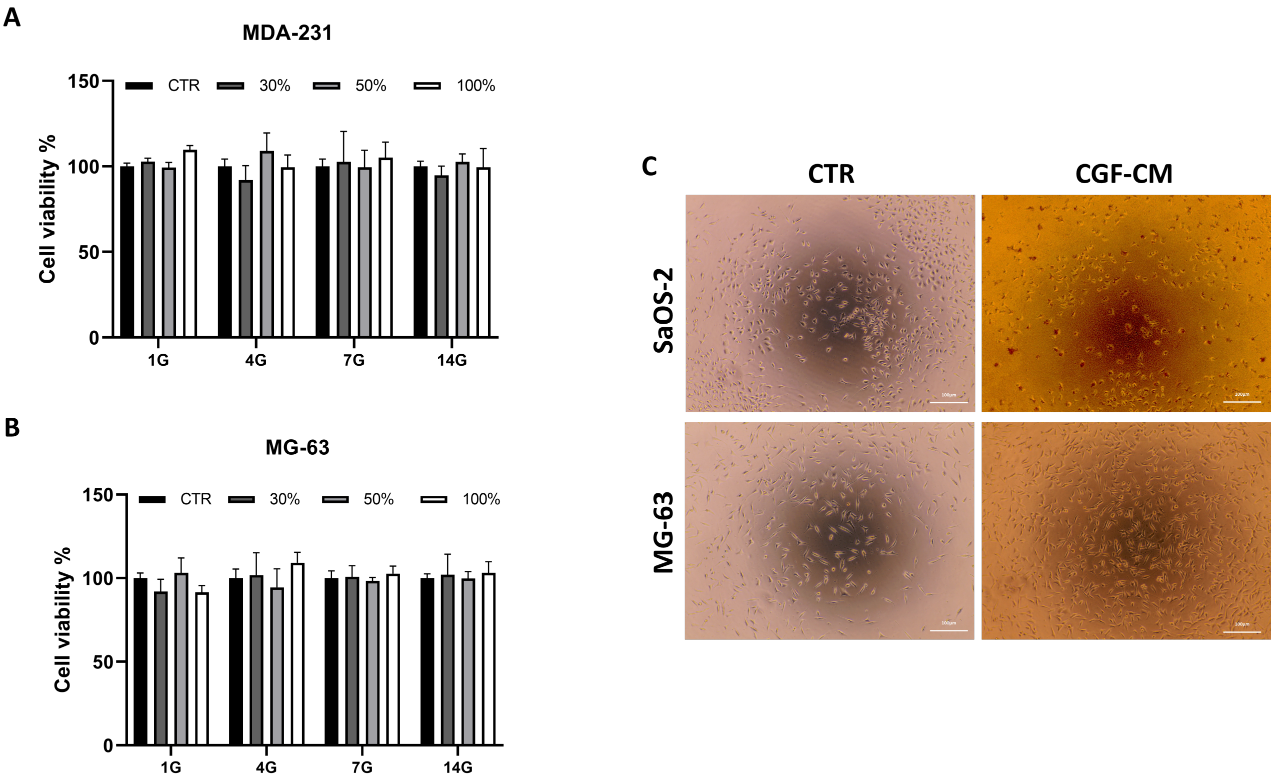

Figure S1: Cell viability was assessed by MTT assay. A) MDA-231 and B) MG-63 were treated with concentrations of CGF-CM ranging from 30% to 100% for indicated times; results were expressed as a percentage of the control cells for each cell lines. Data are presented as the mean ± SD (*n* = 3) and experiments were repeated three times independently. C) Phase-contrast micrographs of cells treated with 30% CGF-CM for 4 days showed alteration in morphology in SaOS-2 but not in MG-63 cells. Images are representative of five independent experiments. Scale bar 100μm.

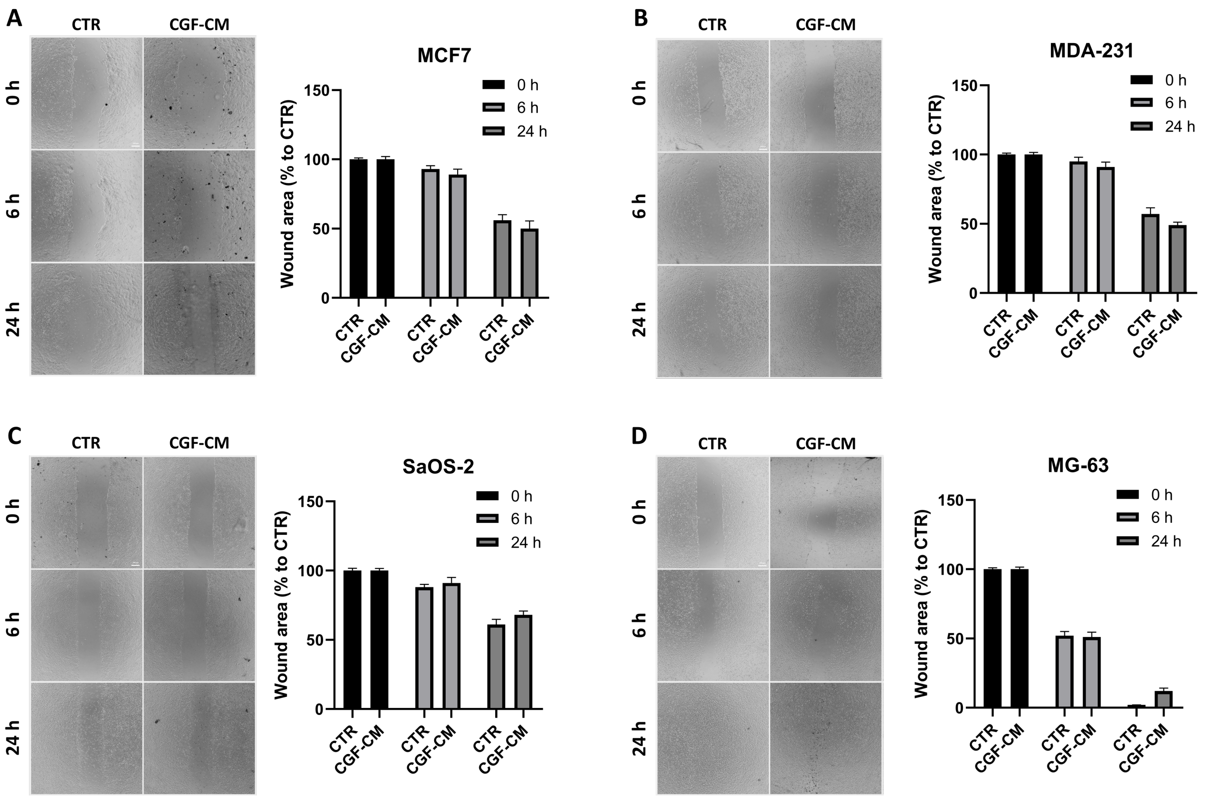

Figure S2: Wound healing assay (scratch assay) in MCF7, MDA-231, SaOS-2 and MG-63 cells treated with 30% CGF-CM for and control cells. Representative pictures of the scratch assay at 0, 6, and 24 h with bar graph illustrating percentage wound area at indicated time points during the scratch wound assay. Scale bar = 200 µm.

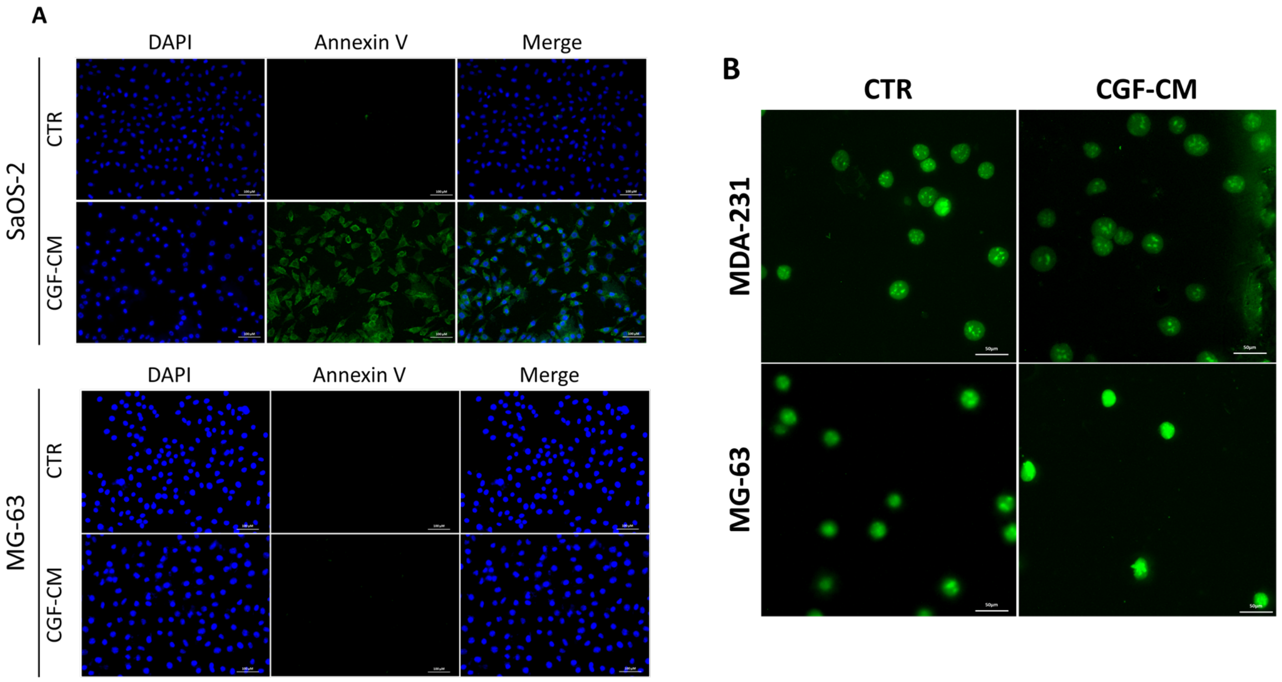

Figure S3: A) Fluorescent micrographs of DAPI and Annexin V staining in SaOS-2 and MG-63 cells treated with 30% CGF-CM for 4 days and control cells. B) Comet assay was performed in untreated MDA-231 and MG-63 cells (CTR), and cells treated with 30% CGF-CM for 4 days.

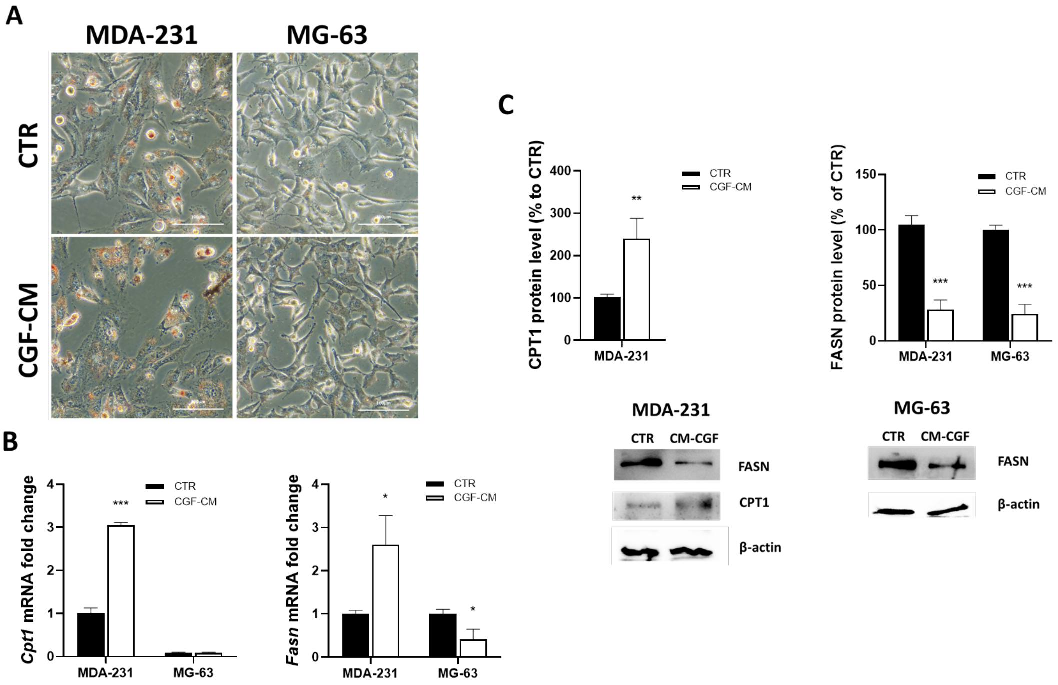

Figure S4: A) The micrographs depict the lipid droplet accumulation, visualized by Oil Red O staining, in cells treated with 30% of CGF-CM for 48h and control cell. The bars in micrographs correspond to 100 µm. Images are representative of three independent experiments. Indicated cell lines were treated with 30% CGF-CM or incubated in DMEM low glucose (CTR) for 48h and mRNA was quantified by Real-time PCR. *Gapdh* was used as a housekeeping gene for normalization. Results are expressed as mean ± SD; experiments were repeated three times independently (n = 3). * p< 0.05 and *** p<0.001 for each cell line compared with its own control. C) Expression protein levels in total protein extracts of each cell lines treated with 30% CGF-CM or incubated in DMEM low glucose (CTR) for 48h. The content of the respective protein was quantified by densitometric analysis and expressed as percentage with respect to own control cells. β-actin was used for normalization. Results are expressed as mean ± SD; experiments were repeated three times independently (n =3). ** p< 0.01 and *** p<0.001 for each cell line compared with its own control.
